# The impact of urban fires on public health

**DOI:** 10.2471/BLT.25.020525

**Published:** 2025-05-01

## Abstract

The impact of fires in urban areas is largely neglected in public health discourse addressing resilient cities. A handful of activists and researchers are trying to change that, Gary Humphreys reports.

When Danielle Antonellis stepped into the charred remains of Grenfell Tower in London, she wasn’t just walking into the aftermath of one of the United Kingdom of Great Britain and Northern Ireland’s worst residential fires, she was entering a defining moment in her career.

A fire safety engineer, Antonellis had been called in to support the provision of expert testimony in the inquiry that followed the fire in 2017. “Walking into the tower was a disturbing experience,” she recalls. “Many people just froze in shock, but focusing on the technical aspects of the fire provided a kind of psychological shield.”

The shield, however, didn’t obscure the bigger picture. The Grenfell fire, which claimed 72 lives, revealed systemic failures in building regulations and fire safety policies. But it also spotlighted something deeper: how unequal fire risk is. 

“The majority of the residents were from lower socioeconomic backgrounds, including working-class families and immigrants, and the inquiry exposed significant concerns about how social housing is managed and maintained,” says Antonellis. “I came away from Grenfell convinced that exposure to fire risk is partly a function of poverty.” 

That realization planted the seed that became Kindling, a non-profit organization founded by Antonellis in 2020 with a mission to bring fire safety to communities poorly served by existing municipal systems. 

An important part of that mission was to research the causes of urban fires, particularly those impacting communities in informal and humanitarian settlements. And the more Antonellis researched, the more she saw how neglected the topic was. “It was clear that people were just not paying attention,” she says.

David Rush takes a similar view. A senior lecturer in structural engineering at the University of Edinburgh, Scotland, Rush takes a particular interest in fire and its impact on structures and residents, and like Antonellis, thinks the topic doesn’t get the attention its impact on public health warrants. 

“Averaged out over a ten-year period, fire and burns cause more death and disability than any other natural hazard, including hazards such as floods and earthquakes,” Rush says.

The exact burden on public health imposed by fire remains open to question due to the lack of data and research in this area, but there are some indicative studies. 

One example is the study published in the December 2019 issue of the *British Medical Journal* which, drawing on data from the 2017 Global Burden of Disease Study, estimated that there were around nine million new injuries and 121 000 deaths due to fire, heat and hot substances in 2017. The World Health Organization estimates that around 180 000 deaths annually are caused by burns.

“Fires and burns cause more death and disability than any other natural hazard.”

David Rush

Rush is quick to point out that the health consequences are not limited to fire’s immediate physical impact. “You also have to factor in the environmental fallout of fire: contaminated water, toxic air and degraded soil quality,” he says, adding that lost earnings, destroyed identification documents, disrupted education, and immediate and long-term psychological trauma should also be taken into account.

Despite the significance of the disease burden, fire rarely figures in global disaster reports such as the annual *World disaster reports* published by the International Federation of Red Cross and Red Crescent Societies. Similarly, there is no specific reference to fire in the United Nations sustainable development goals targets as they relate to risk reduction and resilient cities. 

WHO has done some work in this area. For example, fire prevention figures in *Urban planning, design and management approaches to building resilience – an evidence review, *published in 2022. However, no mention is made of fire in the *Framework for strengthening health emergency preparedness in cities and urban settings,* published by WHO in 2021. 

So why is fire neglected? Professor Rory Hadden, the Rushbrook Lecturer in Fire Investigation at the University of Edinburgh, believes that, unlike earthquakes and floods, the burden of fire may be overlooked because fires tend to be on a smaller scale. 

“Big ones do happen,” he says, “notable examples include the March 2021 fire that swept through the Balukhali refugee camp in Cox’s Bazar, Bangladesh. That fire raged for eight hours, destroying more than 17 000 homes, killed at least 15 people, injured hundreds more and displaced over 45 000 Rohingya refugees; or the recent fires in Los Angeles, United States of America, which destroyed over 16 000 structures and killed 30 people; but generally speaking they are smaller, lower impact but highly frequent occurrences.” 

Francois Petousis agrees. Co-founder and CEO of Lumkani, a social enterprise that has its roots in the University of Cape Town, South Africa’s innovation hub. “The City of Cape Town responded to an average of three fires per day in informal settlements between January 2009 and October 2021,” he says. “Fires happen all the time.”

Hadden also thinks that urban fires may be neglected because they do not – generally speaking – result from natural causes. The perception is that someone causes them. “Rather like traffic accidents, fires are seen as related to behaviours. Of course, that does not stop people talking about road safety as a public health issue, and encouraging seat-belt use,” he points out.

For Hadden, and indeed everyone interviewed for this article, dismissing fires as somehow people’s fault and advocating for behaviour change does not constitute an adequate public health response. “Addressing fire, much like addressing road traffic death and injury, requires comprehensive evidence-based interventions that include changes to the built environment, especially in lower-income settings,” Hadden says.

An estimated 95% of fire-related deaths and injuries occur in low- and middle-income countries. This is attributed not only to limited access to health care but also to the nature of structures in poorer urban areas, especially in informal settlements. 

"Around one billion people globally – one in four urban residents – reside in informal settlements. In these areas, homes are frequently constructed from flammable materials and are densely packed," says Rush, pointing to the fire at Cox’s Bazaar as an example, where closely packed bamboo and tarpaulin shelters were assessed to be aggravating factors.

Rush has made a close study of fire in informal settlements, notably as a co-investigator in the IRIS-Fire (Improving the Resilience of Informal Settlements to Fire) project. His research focuses on identifying, mapping and modelling the risks in informal settlements concerning both fire ignition and spread. 

Hadden’s focus is on the margins of urban areas. Because, as he points out, urbanization affects not only the city itself but also the surrounding rural areas. 

"As people move into cities, they often abandon rural practices that reduced fuel loads, such as wood clearing and controlled burns, leading to significant fuel accumulation," he says. Hadden also points out the vulnerability of city peripheries, where housing is often built in areas where fuel has accumulated, Los Angeles being a case in point.

While focusing on the physical aspects of fire management, Hadden remains acutely aware of the socio-economic determinants at work and believes that effective responses must involve community engagement.

Antonellis shares this view and, in collaboration with the Fire Safety Research Institute (an organization dedicated to advancing fire safety through scientific research and education), launched Kindling FLAMES (Fire Risk Reduction through Learning, Amplification, Mobilization and Empowerment) in April 2024 to address both sides of the issue, integrating scientific research and community engagement. 

She recently completed a five-month field operation focused on fire risk in the informal settlement of Overcome Heights near Muizenberg in Cape Town. Among the key findings of that project was the way fire risk varied as a function of community activity. “Festivals saw a big increase in fires as did weekends, and drinking was a key factor,” she notes. She launched awareness-raising and behaviour change initiatives that included setting up community schemes, whereby people prepared meals for neighbours who were going out of the community to bars. “The initiative is already having an impact,” she says.

Others are taking a more technological approach. Petousis’ Lumkani, for example, produces low-cost networked fire detectors specifically designed for informal settlements. The detectors are triggered by sudden increases in heat rather than smoke to limit false alarms. 

Crucially, they trigger all neighbouring alarms to create a community-wide call to action, simultaneously sending out text messages to the broader community. This enables communities to respond before the fire spreads. To date, the technology has been installed in over 100 000 homes across South Africa, Kenya and Bangladesh. “A third-party study shows that the detectors have helped to limit the spread of fire in over 94% of fire events,” Petousis says. 

For Antonellis, the Lumkani heat detector solution is emblematic: “Fire is a community problem, and unless we confront it with the seriousness it deserves we’ll continue to see preventable tragedies.”

**Figure:**
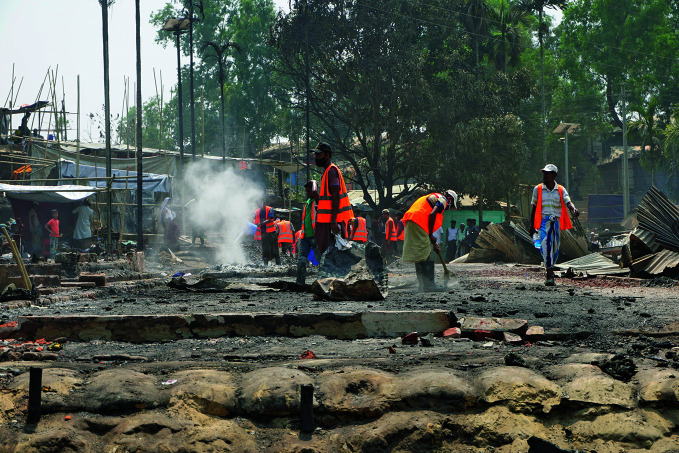
The aftermath of a fire in the Balukhali refugee camp, Cox’s Bazar, Bangladesh, March 2021

WHO/Tatiana Almeida

**Figure:**
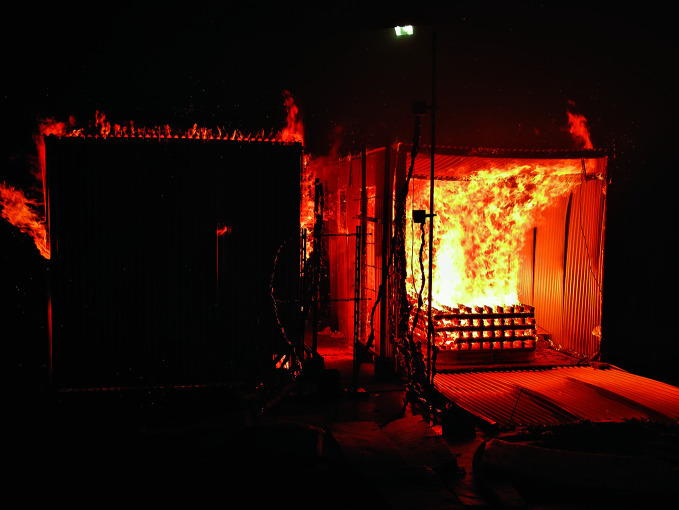
IRIS-Fire Project structural test

Edinburgh University

